# The epigenetic modification of DNA methylation in neurological diseases

**DOI:** 10.3389/fimmu.2024.1401962

**Published:** 2024-09-23

**Authors:** Linke Li, Rui Chen, Hui Zhang, Jinsheng Li, Hao Huang, Jie Weng, Huan Tan, Tailin Guo, Mengyuan Wang, Jiang Xie

**Affiliations:** ^1^ The Center of Obesity and Metabolic Diseases, Department of General Surgery, The Third People’s Hospital of Chengdu and The Affiliated Hospital of Southwest Jiaotong University, Chengdu, China; ^2^ College of Medicine, Southwest Jiaotong University, Chengdu, China; ^3^ Department of Stomatology, The Third People’s Hospital of Chengdu and The Affiliated Hospital of Southwest Jiaotong University, Chengdu, China; ^4^ College of Materials Science and Engineering, Southwest Jiaotong University, Chengdu, China; ^5^ Key Laboratory of Drug Targeting and Drug Delivery of Ministry of Education (MOE), Key Laboratory of Birth Defects and Related Diseases of Women and Children of Ministry of Education, West China Second University Hospital, West China School of Pharmacy, Sichuan University, Chengdu, China; ^6^ Department of Pediatrics, Chengdu Third People’s Hospital, Chengdu, China

**Keywords:** epigenetic regulation, DNA methylation, neurological diseases, ICF syndrome, multiple sclerosis, Rett syndrome, Alzheimer’s disease, Parkinson’s disease

## Abstract

Methylation, a key epigenetic modification, is essential for regulating gene expression and protein function without altering the DNA sequence, contributing to various biological processes, including gene transcription, embryonic development, and cellular functions. Methylation encompasses DNA methylation, RNA methylation and histone modification. Recent research indicates that DNA methylation is vital for establishing and maintaining normal brain functions by modulating the high-order structure of DNA. Alterations in the patterns of DNA methylation can exert significant impacts on both gene expression and cellular function, playing a role in the development of numerous diseases, such as neurological disorders, cardiovascular diseases as well as cancer. Our current understanding of the etiology of neurological diseases emphasizes a multifaceted process that includes neurodegenerative, neuroinflammatory, and neurovascular events. Epigenetic modifications, especially DNA methylation, are fundamental in the control of gene expression and are critical in the onset and progression of neurological disorders. Furthermore, we comprehensively overview the role and mechanism of DNA methylation in in various biological processes and gene regulation in neurological diseases. Understanding the mechanisms and dynamics of DNA methylation in neural development can provide valuable insights into human biology and potentially lead to novel therapies for various neurological diseases.

## 1 Introduction

Neurological diseases are multifactorial disorders caused by intrinsic and extrinsic factors, such as genetics, epigenetics, age and environmental factors, which result in cognitive dysfunction and behavioral disorders (1, 2). With advancing research, neurological diseases are increasingly recognized as complex, multifactorial conditions involving the interplay between neurodegeneration, neuroinflammation, and neurovascular events (3, 4). The neurovascular system (NVS), composed of neurons, glial cells, and vascular cells, is crucial for maintaining brain function by regulating blood-brain barrier (BBB) permeability and cerebral blood flow (CBF) (5, 6). Damage to the BBB can trigger neuroinflammation, while the neurovascular unit (NVU), comprising the BBB and activated microglia, is crucial in the development of neuroinflammation (6). Despite the close functional interdependence of the nervous and vascular systems, the exact mechanisms by which neurovascular dysfunction leads to neurodegeneration remain to be fully elucidated (6). However, it is also essential to consider the roles of genetic and neurodevelopmental factors in these conditions. Genetic factors encompass mutations or variations in specific genes that can contribute to an individual’s susceptibility to certain diseases (7). On the other hand, neurodevelopmental disorders are characterized by abnormalities in brain development processes, which can profoundly affect brain structure and function (8). Neurodegenerative diseases entail the gradual decline of nerve cells and their functionalities, such as Alzheimer’s disease (AD), Huntington’s disease (HD), and Parkinson’s disease (PD) (9). When there is a disruption or blockage in the blood vessels supplying the brain, it can lead to various vascular disorders like stroke and vascular dementia (10). Inflammatory diseases, including multiple sclerosis (MS) and meningitis, are characterized by inflammation and damage to the nervous system (11). Functional disorders, such as epilepsy and migraine, involve abnormal electrical activity in the brain or dysfunction of specific neural circuits (12). Neurological diseases impose a significant burden on affected individuals as well as healthcare systems worldwide (13, 14). Since 1990, there has been a significant increase in mortality attributed to neurological diseases, with a rise of approximately 37% from 6.87 million to 9.40 million. According to the Global Burden of Disease Study, the total disability-adjusted life years (DALYs) worldwide have increased by about 7% from 233.4 million to 250.7 million. Neurological diseases had become the leading cause of DALYs, second only to cardiovascular diseases, and a major global determinant of mortality (15).

Epigenetic mechanisms, such as DNA methylation, non-coding RNA regulation, and histone modifications, are involved in these processes (16, 17). Among them, DNA methylation is indispensable for gene expression regulation, genomic integrity, genomic imprinting, and X chromosome inactivation (18). However, environmental factors, aging and diseases could affect DNA methylation (19). During and after DNA replication, DNA methylation is a crucial process in the dynamic environment of chromatin reorganization (20). Alterations in DNA methylation patterns can influence gene expression and cellular processes within the cardiovascular and neurological systems, contributing to the pathogenesis and progression of these diseases (21, 22).

In the context of neural development, DNA methylation has emerged as a crucial mechanism in regulation of neural stem cell proliferation, synaptic plasticity, and neuronal repair (23, 24). In many neurological diseases, comprising both neurodegenerative and neurodevelopmental disorders, alterations in DNA methylation occur at specific genomic regions, including gene promoters, enhancers and CpG islands (25). These changes can lead to dysregulation of gene expression and disruption of normal cellular processes. For example, DNA hypermethylation in the promoters of genes associated with memory formation and synaptic plasticity has been observed in AD (26). This altered methylation pattern may contribute to the cognitive decline and neurodegeneration in AD. In PD and HD, changes in DNA methylation have also been reported (27, 28). These alterations can have profound effects on gene expression associated with dopaminergic signaling, mitochondrial function, and neuroinflammation, which are key processes implicated in complex mechanisms of these diseases (29). Understanding the specific DNA methylation changes and their functional consequences in different neurological disorders can provide insights into disease mechanisms and potential therapeutic targets.

## 2 DNA methylation

DNA methylation is catalyzed by DNA methyltransferases (DNMTs) with the transferring a methyl group to the cytosine ring within both CpG and non-CpG dinucleotides (20, 30). CpG dinucleotides are regions in DNA where a cytosine is followed by a guanine, often found in clusters called CpG islands (31). Non-CpG dinucleotides refer to any other dinucleotide sequence without a cytosine followed by a guanine (32). Although CpG methylation is well-studied, it has been discovered that non-CpG methylation, particularly at CpH dinucleotides (H = A, T, or C), can also occur in specific cell types or stages of development (33). Non-CpG methylation has been observed to be more abundant in pluripotent stem cells, neurons, and certain cancers, suggesting its involvement in cell fate determination and disease development (34).

**Figure 1:**
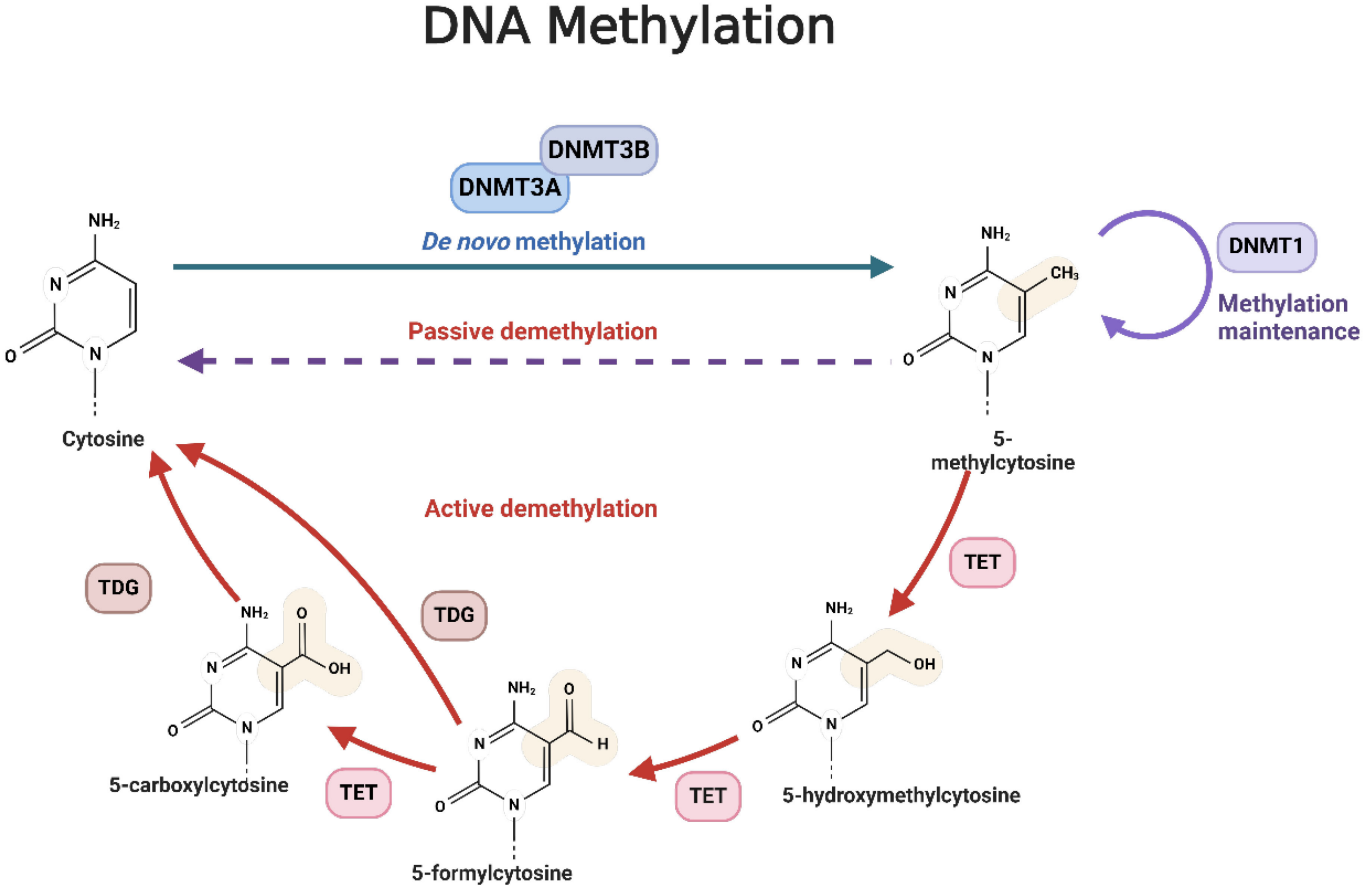
The mechanism of DNA methylation. The methyl groups are transferred from the S-adenosine-L-methionine (SAM) molecule to the position of cytosine C5 under the action of methyltransferase, especially DNMT3a/3b. 5-methycytosine is obtained after transferring. These methylated methyl groups can regulate gene expression by altering or modifying gene expression. DNA demethylation is the process by which a methyl group (-CH3) is removed from a DNA molecule. There are two primary mechanisms of DNA demethylation: active demethylation and passive demethylation. The first pathway involves the ten-eleven translocation (TET) family of enzymes, which can oxidize the methyl group on the cytosine base of DNA. These oxidized forms of cytosine can then be further modified and removed from the DNA molecule through the base excision repair (BER) pathway. The second pathway involves DNA demethylase enzymes which can directly remove the methyl group from the cytosine base. These enzymes can recognize and bind to methylated cytosine residues, and then catalyze the removal of the methyl group through a series of enzymatic reactions.

There are three main DNMTs involved in DNA methylation: DNMT1, DNMT3A, and DNMT3B (35). DNMT1 maintains established DNA methylation patterns, while DNMT3A/3B are involved in *de novo* DNA methylation (36). DNA demethylation encompasses the removal of a methyl group (-CH3) from DNA and proceeds through two fundamental mechanisms: active demethylation, which implicates the TET enzyme family catalyzing the oxidation of the methyl group on cytosine, and passive demethylation, where DNA demethylase enzymes directly excise the methyl group from cytosine (**Figure 1**) (37–39). The TET protein family functions as a dioxygenase dependent on α-ketoglutarate (α-KG) and Fe^2+^, with its catalytic center composed of a Cys-rich domain near the C-terminus and a double-strand α-helix (DSBH) domain (40). In mammals, the TET family consists of TET1, TET2, and TET3 (40). Both TET1 and TET3 feature a CXXC zinc finger domain at their N-terminus, which facilitates their binding to chromatin and recognition of DNA methylation regions (40). TET1 specifically binds to target DNA through its CXXC domain, which uniquely interacts with unmethylated CpG dinucleotides, typically occurring at gene promoters or enhancer regions crucial for gene expression regulation (41, 42).

Once TET1 is bound to DNA, it utilizes its oxidase activity to oxidize 5-methylcytosine (5mC) through a multi-step process (43). Initially, 5mC is converted to 5-hydroxymethylcytosine (5hmC), representing the first stage of demethylation (43). TET1 then further oxidizes 5hmC to 5-formylcytosine (5fC), and subsequently, which is subsequently oxidized to 5-carboxylcytosine (5caC) (43). The final step involves the removal of 5caC from the DNA, which is carried out by DNA repair mechanisms (44). Specifically, the base excision repair (BER) pathway employs enzymes like thymine DNA glycosylase (TDG) to recognize and excise 5caC, creating a basic site (44). The DNA repair system then inserts a normal cytosine to complete the repair process (44). Through these actions, TET1 lowers DNA methylation levels and regulates gene expression, impacting various cellular processes (45).

## 3 Recent advances in omics approaches for DNA methylation studies in neurological diseases

Omics approaches have provided unprecedented insights and new perspectives in the study of DNA methylation in neurological diseases. By employing these omics approaches, researchers can systematically analyze and quantify DNA methylation changes in neurological diseases, offering new insights and strategies for disease diagnosis, treatment, and prevention. Here are some key omics methods and their recent advancements in this field.

### 3.1 Whole-genome bisulfite sequencing

WGBS is a preeminent technique for interrogating DNA methylation across the genome (46). It entails denaturing DNA into single strands and subjecting them to bisulfite treatment, which discriminates between methylated and unmethylated cytosines. Acknowledged as the gold standard for DNA methylation analysis, WGBS has been instrumental in elucidating DNA methylation alterations associated with neurodegenerative disorders such as AD and PD, offering crucial insights into their underlying mechanisms (46).

WGBS has demonstrated its significant value in various studies. For instance, in evaluating the epigenetic regulatory effects of xenon (Xe) in a rat model of stroke, WGBS revealed that Xe modulates the methylation status of several genes associated with neurocognitive recovery, including Gadd45b, DNMT3a, and HDAC9, which are linked to memory and aging, as well as growth factor-related genes like NTrK2 and NGF. Furthermore, Xe was shown to influence the methylation of endogenous β-secretase (BACE1) and low-density lipoprotein receptor-related protein 1 (LRP1), both of which are closely related to amyloid-β (Aβ) production (47).

In another study, WGBS was employed to analyze postmortem brain tissue and blood samples from AD patients and control groups (48). The findings highlighted significant differences in DNA methylation between AD patients and controls, particularly within a candidate imprinting control region (ICR) on chromosome 17, associated with the NLRP1 inflammasome gene. Additionally, a single nucleotide polymorphism (SNP) found in the blood samples further suggested that imprinting abnormalities may be linked to the risk of developing AD, supporting the hypothesis that early-life environmental exposures could increase the risk of AD in later life (48).

In a PD mouse model exposed to fenvalerate (Fen), WGBS was used to assess DNA methylation in the midbrain, revealing differential methylation of the Ambra1 gene, which is associated with PD (49). This differential methylation, characterized by hypermethylation and reduced expression of Ambra1, suggests that Ambra1 plays a pivotal role in PD pathogenesis through its regulation of the mitophagy pathway, a process influenced by DNA methylation (49).

In the context of MS, WGBS was combined with an optimized MHC capture approach to investigate the methylation status of the MHC region in blood samples from 147 MS patients and 129 healthy controls (50). The study identified 132 differentially methylated regions (DMRs) associated with MS, overlapping with known MS risk loci, and found that these methylation changes were linked to specific human leukocyte antigen (HLA) genotypes. Through DNA methylation quantitative trait loci (mQTL) mapping and causal inference testing (CIT), several DMR-SNP pairs were identified, potentially mediating MS risk, underscoring the importance of WGBS in elucidating epigenetic alterations associated with MS (50).

In studies of immune deficiency, centromere instability, and facial anomalies (ICF) syndrome, WGBS revealed a substantial reduction in overall methylation levels, notably in inactive heterochromatin regions, satellite repeats, and transposons (51). Despite these changes, methylation levels remained high in transcriptionally active sites and ribosomal RNA repeats (51). The research also identified a mutation in DNMT3B leading to the mislocalization of H3K4me1 activity, resulting in hypermethylation of active promoters—a finding closely related to the immunodeficiency phenotype of ICF syndrome, particularly in genes involved in B-cell receptor-mediated maturation pathways (51).

WGBS, despite its analytical strengths, faces limitations with low-input samples such as circulating cell-free DNA and single-cell sequencing (46). The conversion of unmethylated cytosines to thymine reduces sequence complexity, impacting sequencing quality, mapping rates, and genome coverage, and increasing costs (46). However, WGBS remains the most comprehensive method for high-resolution mapping of cell type-specific methylation patterns, though its high cost limits wider use.

### 3.2 Reduced representation bisulfite sequencing

RRBS is a cost-effective and sensitive method widely used in neurodevelopmental disorder research, including autism spectrum disorders (52). It efficiently targets CpG-rich regions like promoters and enhancers, providing precise methylation data with reduced sequencing depth (52). Although it covers only 6-12% of genome-wide CpG sites, RRBS is effective for detecting DNA methylation patterns and is well-suited for formalin-fixed samples, making it a promising option for large-scale clinical studies (52).

RRBS has been employed in several studies to analyze DNA methylation changes in AD and related models (53). In one study, researchers used RRBS to investigate genome-wide DNA methylation changes in AD brains, discovering that the CASPASE-4 (CASP4) gene exhibited reduced DNA methylation in AD brains, which was correlated with increased CASP4 expression (54).

In another study, RRBS was utilized to analyze DNA methylation changes in the TG4510 transgenic mouse model, which overexpresses the P301L mutant human tau protein—a common tauopathy model in AD research (55). RRBS results revealed significant DNA methylation differences in brain regions and blood of TG4510 mice, closely associated with transcriptomic features related to tau pathology, providing valuable insights into potential biomarkers for AD (55).

Furthermore, RRBS has been applied to explore the epigenetic landscape of AD. In a study involving 471 brain samples, RRBS was used to measure DNA methylation levels in the temporal cortex (TCX) and cerebellum (CER) (56). This research identified specific CpG sites associated with AD-related neuropathological measures (such as Braak stage, Thal phase, and cerebral amyloid angiopathy scores) and AD-related proteins (e.g., Aβ40, Aβ42, tau, and p-tau) and uncovered region-specific CpG associations between TCX and CER. The findings suggest that while neuropathological and biochemical markers reflect the core pathology of AD, distinct DNA methylation changes are uniquely associated with these markers, revealing diverse epigenetic processes at play (56).

### 3.3 Methylation arrays (e.g., Infinium 450K/850K)

Methylation arrays are high-throughput tools for analyzing DNA methylation across the genome, including CpG islands, promoters, and enhancers (57, 58). Despite their effectiveness, they face limitations such as high costs, complex data interpretation, sensitivity and specificity issues, limited spatial resolution, and restricted coverage to known CpG sites, offering limited insights into the underlying mechanisms of methylation (57).

In AD research, researchers employed methylation arrays alongside PCR-based methylation-sensitive high-resolution melting (MS-HRM) analysis to examine blood DNA from 56 patients with late-onset Alzheimer’s disease (LOAD) and 55 healthy controls (59). The results from both techniques revealed no significant differences in the methylation levels of key DNA repair genes between LOAD patients and controls. This indicates that the hypothesized increase in promoter methylation of these genes in the blood DNA of AD patients is not supported by the current data (59).

Furthermore, in screening for FXS, methylation arrays demonstrated exceptional sensitivity and specificity. The study assessed genome-wide and FMR1-specific DNA methylation in 32 male individuals diagnosed with FXS, including 9 males with mosaic mutations, 5 females with full mutations, and 11 male and 11 female premutation carriers (60). When compared with 300 normal control DNA samples, the methylation array achieved 100% sensitivity and specificity in detecting FXS in male patients, effectively distinguishing those with mosaic methylation defects (60). This finding supports the utility of methylation arrays as a cost-effective and sensitive screening tool for FXS, capable of simultaneously excluding other common differential diagnoses such as Prader-Willi syndrome and Sotos syndrome (60).

### 3.4 Methylation-specific PCR

MSP is a sensitive, cost-effective, and efficient method for detecting DNA methylation, making it suitable for small-scale analyses (61, 62). However, it is limited by its qualitative or semi-quantitative nature, relatively low specificity, the need for precise primer design, and challenges arising from uneven 5mC distribution (62).

In studies of longevity-associated genes, MSP has been employed to analyze the methylation status of SIRT3, SMARCA5, HTERT, and CDH1 promoters in peripheral blood (63). Results indicate that the methylation frequencies of SIRT3, SMARCA5, and CDH1 do not differ significantly among young individuals, elderly individuals, and AD patients, suggesting that methylation of these genes is not related to aging or AD (63). Conversely, the methylation frequency of HTERT is associated with the aging process and is significantly higher in AD patients compared to elderly controls, potentially reflecting AD-related telomeric and immune dysfunctions (63).

Furthermore, MSP has been applied to investigate epigenetic modifications related to PD. In a study of early-onset Parkinson’s disease (EOPD) patients, MSP assessed the methylation status of the SNCA and PARK2 gene promoter regions in 91 EOPD patients and 52 healthy controls (64). The findings reveal that the methylation levels of the SNCA and PARK2 promoters are significantly lower in EOPD patients compared to controls, with SNCA methylation status potentially linked to a positive family history of PD (64). These observations suggest a role for epigenetic modifications in PD.

### 3.5 Methylated DNA immunoprecipitation sequencing

MeDIP-seq is effective for genome-wide identification of highly methylated regions, offering broad coverage and high CpG-level resolution at reduced costs (65, 66). However, it requires highly specific antibodies, still involves relatively high sequencing costs, and is less effective at distinguishing specific methylation regions compared to other methods (66).In a multicenter study conducted in Italy, MeDIP-seq was used to analyze 26 affected and 26 unaffected relatives from 8 MS families (67). The study combined association and aggregation statistics across families to identify 162 differentially methylated regions (DMRs) (67). Through technical validation and biological replication, two hypomethylated regions (linked to the NTM and BAI3 genes) and two hypermethylated regions (located in the PIK3R1 and CAPN13 genes) were confirmed (67). These findings highlight the value of MeDIP-seq in uncovering the epigenetic mechanisms underlying complex diseases.

### 3.6 Pyrosequencing

Pyrosequencing is a highly precise, real-time sequencing-by-synthesis technology used for high-resolution DNA methylation analysis, mutation detection, and genomic research. It detects light signals produced by the release of pyrophosphate during nucleotide incorporation (68, 69). In one study, pyrosequencing was employed to measure DNA methylation levels in blood, analyzing 46 cytosine-guanine sites across 21 genes (including NXN, ABCA7, and HOXA3) to evaluate its diagnostic accuracy for non-invasive detection of late-onset AD (70). Despite its advantages, pyrosequencing has limitations, including a short read length (around 150 base pairs), susceptibility to signal deviations, and a dependency on sample quality and library preparation, which can introduce errors (68). Additionally, sequencing depth can affect data quality, either by missing important mutations or introducing errors (69). While rapid and efficient, these limitations should be considered when selecting the most appropriate sequencing method for specific research needs.

## 4 DNA methylation in neurological diseases

DNA methylation patterns are dynamically reprogrammed with global DNA demethylation followed by *de novo* methylation during early embryonic development (71). In somatic cells, DNA methylation patterns are generally stable due to the action of maintenance DNA methyltransferase (72). Erroneous DNA methylation usually leads to gene silencing by inhibiting the binding of transcription factors or other DNA-binding proteins (73). The factors influencing DNA methylation in neurological diseases are multifactorial and include both genetic and environmental factors (74). Genetic variations in genes encoding DNA methyltransferases (DNMTs) and other proteins involved in DNA methylation machinery can affect the stability and fidelity of DNA methylation patterns (75). Environmental factors, such as prenatal exposure to toxins or stressful events, can also impact DNA methylation patterns in the developing brain and increase the risk of neurological diseases (76).

Aberrant DNA methylation patterns have been observed in various neurological disorders, contributing to changes in gene expression and cellular function (77). Here, we summarize the mechanisms and dynamics of DNA methylation alterations in neurological diseases, such as immunodeficiency, centromeric instability, and facial anomalies (ICF) syndrome, Rett syndrome (RTT), multiple sclerosis (MS), amyotrophic lateral sclerosis (ALS), Alzheimer’s disease (AD), Parkinson’s disease (PD), Huntington’s disease (HD), fragile X syndrome (FXS), and epilepsy.

### 4.1 Immunodeficiency, centromere instability, and facial abnormality syndrome

**Figure 2:**
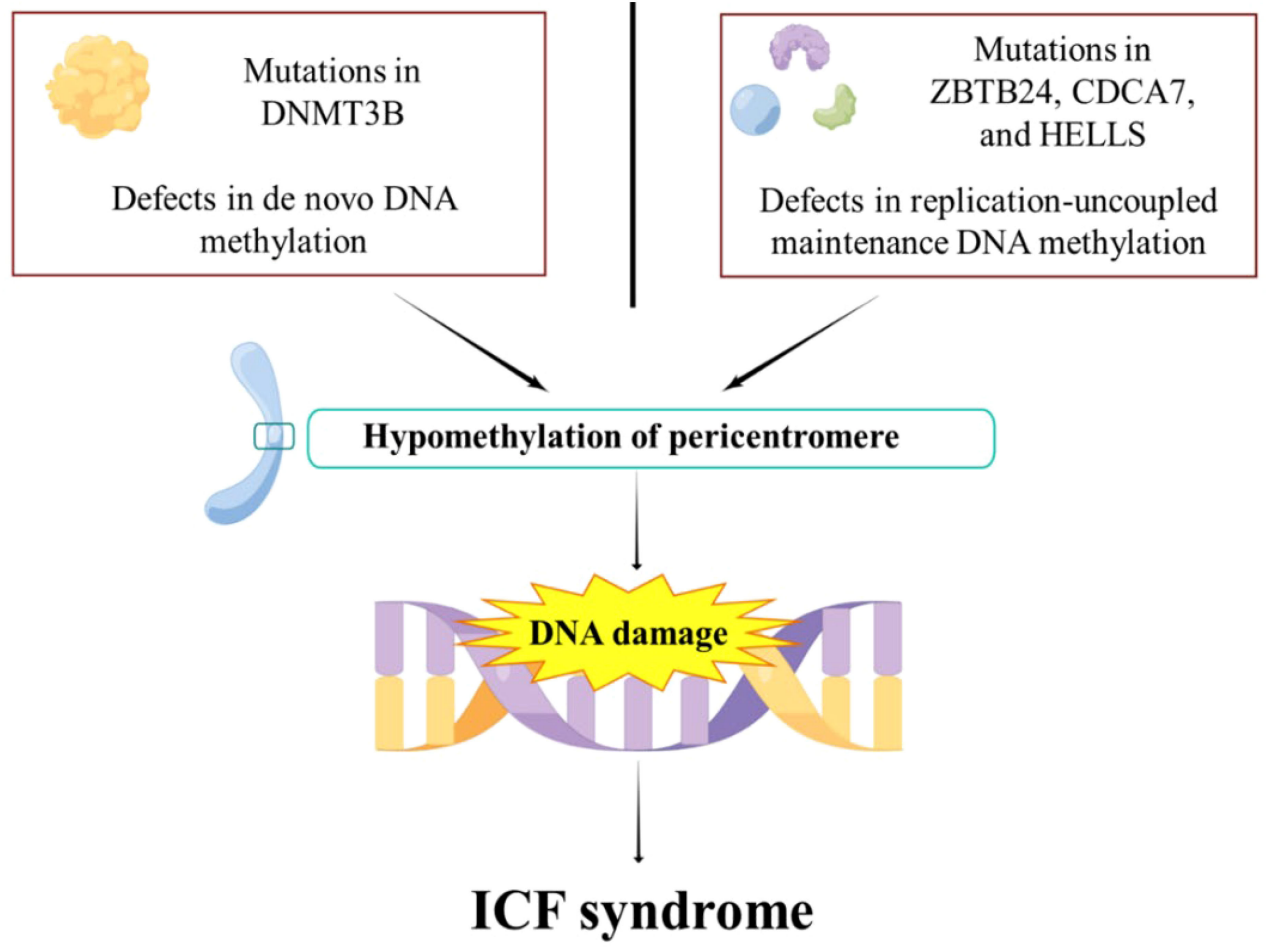
The pathogenesis of ICF syndrome. DNMT3b mutations impair *de novo* DNA methylation. ZBTB24, CDCA7, and HELLS mutations lead to impaired replication-uncoupled maintenance of DNA methylation, resulting in pericentromeric hypomethylation. This subsequently leads to DNA damage, which eventually evolves into ICF syndrome.

ICF syndrome is a rare autosomal recessive genetic disorder characterized by fatigue, facial abnormalities, and cognitive deficits (78). The majority of cases are attributed to mutations in one of five genes: DNMT3b, zinc finger and BTB domain containing gene 24 (ZBTB24), cell division cycle related gene 7 (CDCA7), lymphospecific helicase (HELLS), and ubiquitin like PHD and RING finger domain 1 (UHRF1) (79, 80) (**Figure 2**). However, in some instances, the causative gene remains unknown (81). ZBTB24 acts as a transcription factor targeting CDCA7, and CDCA7 together with HELLS protein forms a chromatin remodeling complex (79). The pathogenic variants in ZBTB24, CDCA7, and HELLS disrupt DNA methylation maintenance unrelated to replication, leading to low methylation in specific regions that rely on CDCA7/HELLS complexes for chromatin remodeling (79). CDCA7 and HELLS, through their chromatin remodeling activity, may promote the localization of Ku80 to double strand break (DSB) sites, thereby supporting non homologous terminal junction (NHEJ) repair (82, 83). This mechanism is associated with significant features of ICF syndrome, namely reduced DNA methylation of satellite repeat sequences near the centromere, leading to chromosomal instability and abnormal condensation of heterochromatin in lymphocytes (36, 84).

Recent studies have shown that the structure of DNMT3b’s methyltransferase domain is more susceptible to mutations associated with ICF syndrome compared to its complex with DNMT3L (85). Efforts to correct DNA methylation abnormalities by targeting DNMT3b have achieved limited success, highlighting the complexity of the disease’s molecular mechanisms (85). In the cellular context, the protein HELLS, a member of the SNF2 ATPase family, is important for new DNA methylation through its interaction with DNMT3b (86). It works alongside CDCA7 in remodeling nucleosomes, which in turn affects DNA methylation (83). Mutations in both HELLS and CDCA7 have been found to disrupt normal DNA methylation patterns, which cause the progression of ICF syndrome (87). Recent findings suggest that ZBTB24 and CDCA7 can indirectly affect DNA methylation via DNMT3b (88). ZBTB24 does not directly bring DNMT3b to specific areas of the genome but seems to work mainly through CDCA7 (89). This highlights the central role of CDCA7 in maintaining normal DNA methylation.

In summary, mutations in DNMT3b, ZBTB24, CDCA7, and HELLS disturb the normal process of DNA methylation, which is critical to the pathogenesis of ICF syndrome (90). In the clinical management of ICF syndrome, current protocols largely rely on immunoglobulin replacement therapy coupled with prophylactic antibiotic interventions (91, 92).

### 4.2 Rett syndrome

RTT is a multifaceted neurodevelopmental disorder characterized by cognitive delays, developmental impairments, respiratory complications, motor dysfunctions, epilepsy, and an elevated risk of sudden death (93, 94). Numerous studies have identified mutations in the methyl-CpG binding protein 2 (MECP2) gene as the primary etiology of most RTT cases. Oxidative stress has been identified as a critical factor in RTT pathogenesis (95). The loss or dysfunction of MECP2 results in elevated levels of reactive oxygen species (ROS), leading to DNA damage, protein oxidation, and lipid peroxidation (96, 97). As a severe X-linked neurodevelopmental disorder, RTT predominantly affects females, with the majority of cases resulting from MECP2 mutations both within and outside the methyl-CpG binding domain (MBD) (98). Approximately 70% of RTT cases are attributed to eight specific missense and nonsense mutations, including R106W, R133C, R168X, R255X, R270X, R294X, R306C and T158M) (99), while about 15% result from large deletions within the MECP2 gene. These mutations lead to the loss or dysfunction of MECP2, disrupting DNA methylation patterns and subsequent gene expression, ultimately affecting normal brain development and function (100–102).

The MECP2 protein comprises several functional domains, including the N-terminal domain (NTD), MBD, interdomain (ID), transcriptional repression domain (TRD), and C-terminal domain (CTD) (103, 104). TRD and NID domains are essential for recruiting gene repressors, such as the deacetylase HDAC3, while the ID domain stabilizes the structure and binding ability of the MBD (105). MECP2 also contains three AT-hook motifs in the TRD, CTD, and ID, which enhance its ability to bind AT-rich DNA sequences. Mutations in the MBD of MECP2, affecting its DNA-binding affinity, can be classified into three categories: those causing significant reduction (e.g., L110V, S134C, P152R, D156E), moderate increase (e.g., A140V, R111G), and minimal or slight effects (e.g., F155S, R106Q, R106W, R133C, R133H) on MECP2’s DNA-binding affinity (34, 106, 107). These structural domains and motifs enable MECP2 to interact with specific DNA sequences, functioning as both a transcriptional activator and repressor depending on the methylation patterns and cellular context (108). MECP2 selectively repress the expression of long genes in neurons by binding to CA methylation sites, contributing to the cellular anomalies observed in RTT (109). MECP2 also acts as an activator by interacting with CREB (cAMP response element-binding protein), playing a role in neuronal maturation. The delayed onset of RTT symptoms is believed to be associated with the postnatal accumulation of methylated CpA dinucleotides (mCpA) and MECP2 binding to these sites, contributing to progressive neurodevelopmental abnormalities (110).

Using murine models, researchers have highlighted the key role of MECP2 in brain function and development. Constitutive MECP2 knockout (KO) mice, designed to study MECP2 loss, exhibit severe neurological symptoms such as uncoordinated gait, hindlimb clasping, and irregular breathing after an initial period of normal development (111, 112). These mice exhibit decreased brain volume and smaller, more densely packed neurons in the hippocampus, cortex, and cerebellum (113, 114). Further investigations using a conditional knockout approach with the Nestin-Cre transgene to ablate MECP2 expression specifically in the brain during embryonic development revealed phenotypes similar to those of constitutive KO mice, indicating that the observed abnormalities stem from neuronal dysfunction (115). Moreover, transgenic mice overexpressing MECP2 recapitulate numerous behavioral phenotypes observed in R MECP2 KO mice, highlighting the necessity of precise MECP2 level regulation for proper central nervous system function. Both the absence and overexpression of MECP2 lead to neurofunctional deficits akin to those observed in RTT patients, emphasizing the need for careful control of MECP2 expression (116, 117).

However, the relationship between MECP2 and RTT still poses many unresolved questions and challenges. For example, selecting an efficient delivery vector, ensuring it reaches a sufficient number of cells, preventing the newly implanted gene from becoming inactive, and managing any adverse effects from the added MECP2. Current research explores various viral vectors, such as lentiviruses, retroviruses, and adeno-associated viruses (AAVs), for gene delivery (118). Initial experiments utilizing AAVs to deliver MECP2 into mice lacking this protein have shown promising results. Recent studies demonstrated that the miRNA-Responsive Autoregulatory Element (miRARE) effectively reduced toxicity in self-complementary AAV9 miniMECP2 gene therapy in mice (119). TSHA-102, the human-ready version, showed significant improvements in respiration, weight, survival, and motor function in Mecp2−/y knockout mice, supporting its advancement to clinical trials for RTT (NCT05606614). These studies demonstrated that the reintroduction of MECP2 led to alleviated motor impairments and improved spontaneous movement in these mice (120).

Ongoing research on MECP2 delivery methods and therapeutic approaches, including gene therapy and novel pharmacological agents, emphasizes MECP2’s pivotal role in DNA methylation and its therapeutic potential for RTT. Understanding the intricate relationship between MECP2, DNA methylation, and cellular processes is crucial for unraveling the molecular mechanisms underlying RTT (121). Further research is needed to fully elucidate how mutations in MECP2 disrupt its function and contribute to the development of RTT. Additionally, further exploration is needed to understand the correlation between DNA methylation patterns and the clinical manifestations of these conditions.

### 4.3 Multiple sclerosis

Multiple sclerosis (MS), an autoimmune-mediated neurodegenerative disease, which is characterized by progressive neurological dysfunction with a notably higher incidence among young adults (122, 123). Within the broader context of neurodegenerative disorders, MS is unique for its distinctive alterations in DNA methylation landscapes (124). DNA methylation changes in genes associated with immune response, inflammation, and myelination have been observed in MS (125, 126). Oxidative stress is a critical factor in the pathogenesis of MS. This oxidative damage exacerbates the demyelination process and contributes to neuronal injury (127). Additionally, the etiology of MS involves complex interactions between genetic, non-genetic, and random factors, making research into causative treatments still in its early stages (127). Genome-Wide Association Studies (GWAS) have proven to be critical tools in unraveling the etiology of MS (127). By comparing disease cases with controls, GWAS has identified over 200 genetic loci associated with MS (127). Advances in methodology, increased sample sizes, and improvements in statistical techniques have greatly enhanced our understanding of MS genetic structure (128).

Current research has expanded beyond the previously emphasized Human Leukocyte Antigen DR 1 (HLA-DRB1) to uncover several new key genetic risk factors, including TET2 (10-11 Translocation 2), Foxp3 (Forkhead Box P3), ATXN1 (Ataxin-1), RP11-326C3.13, TNFSF14, Dysferlin (DYSF), and Zinc Finger Protein 638 (ZNF638) (129–133). These discoveries offer new insights into the genetic background and etiology of MS (**Table 1**). Compared to healthy controls, MS patients show significantly downregulated expression of TET2 in peripheral blood mononuclear cells (PBMCs), correlating with abnormal methylation patterns in the promoter regions of TET2 and DNMT1 (134). These abnormal methylation patterns contribute to the epigenetic dysregulation observed in MS pathology (81). Additionally, hypermethylation of the Foxp3 gene promoter in T cells results in reduced Foxp3 expression, impairing Treg cell function and disrupting immune homeostasis, thereby exacerbating MS progression (83, 85). GWAS have identified RP11-326C3.13 and TNFSF14 as consistently associated with multiple omics layers (132). Furthermore, a mutation associated with rapid disease progression in MS has been identified between the previously unlinked genes Dysferlin (DYSF) and Zinc Finger Protein 638 (ZNF638) (133). DYSF is involved in cell damage repair, while ZNF638 helps control viral infections, suggesting that this mutation may be directly related to disease progression.

**Table 1:** DNA methylation and multiple sclerosis.

Gene/ protein	Function	Mechanism	Reference
TET 2	Contributing to the epigenetic dysregulation in MS pathology	Regulating the promoter regions of TET2	(134)
HLA-DBR1*15:01	A genetic risk factor for MS	Key for antigen presentation to CD4+ T cells	(235)
HLA-DRB1*07:01	Increasing MS risk	Affecting CD4+ T cell activation	(236)
HLA-DRB1 *11	Affecting MS susceptibility	Influencing antigen presentation to CD4+ T cells and immune response	(236)
Foxp3	Exacerbating MS progression	Hypermethylation of the Foxp3 gene promoter in T cells, impairing Treg cell function	(237)
ATXN1	Contributing to MS pathogenesis	Hypomethylation of the ATXN1 gene in B cells	(124)

The complexity of MS pathology is highlighted by the dynamic interactions between various cellular components, including the immune system, glial cells, and neurons (135). Studies using animal models such as experimental autoimmune encephalomyelitis (EAE) in mice and marmosets, as well as analyses of human cerebrospinal fluid (CSF) and peripheral blood samples, have provided insights into these interactions (136, 137). For instance, B cells from MS patients exhibit hypomethylation and have a higher number of differential methylation sites (DMPs) compared to T cells. In B cells, hypomethylation of the ATXN1 gene correlates with MS risk variants and leads to elevated levels of ATXN1 mRNA, potentially contributing to MS pathogenesis (124). These findings suggest that DNA methylation changes in specific genes, such as ATXN1, in B cells may play a role in MS development (124). Additionally, cell type-specific differentially methylated positions (csDMPs) research indicates that over 60% of csDMPs are predominantly hypermethylated in B cells, while csDMPs in monocytes are more evenly distributed (124). Significant differential methylation effects in the HLA regions highlight notable variations between cell types, with strong signals originating primarily from monocytes and B cells, and to a lesser extent, from T lymphocytes (124). These findings underscore the complex interplay among various cell types and their contributions to MS pathology (137).

Recent epigenetic research in MS has indeed identified several promising therapeutic targets. DNA Methylation inhibitors, such as 5-azacytidine and decitabine, can modulate the immune response and suppress the abnormal activation of immune cells by regulating aberrant methylation patterns in MS (73). However, 5-azacytidine is not without limitations. Despite its potential, 5-azacytidine has demonstrated significant toxicity in multiple mouse and cell models. This toxicity raises concerns about its safety and long-term use in clinical settings (138, 139). TET2 is crucial for maintaining proper DNA methylation patterns, and its downregulation is associated with MS pathology. Compounds targeting TET2, such as 5-aza-2’-deoxycytidine (Decitabine), aim to restore normal methylation processes and potentially ameliorate disease symptoms (140, 141). Similarly, Foxp3 is essential for regulatory T cell (Treg) function, and its hypermethylation impairs immune regulation in MS. Compounds targeting Foxp3, such as Trichostatin A, G9a inhibitors, and Resveratrol, aim to correct these epigenetic disruptions and restore Treg cell function, which could be pivotal in managing MS. Immunotherapies targeting T regulatory (Treg) cells and HLA-DRB1 have shown early promise, but require rigorous evaluation in randomized clinical trials to definitively establish their safety and efficacy (142).

Understanding DNA methylation’s role and its interaction with risk factors in different cell types involved in MS is crucial for unraveling the mechanisms of this complex neurological disorder and identifying potential therapeutic targets. Further research is required to decode the intricate relationships between DNA methylation, gene expression, and cellular components in MS pathogenesis.

### 4.4 Amyotrophic lateral sclerosis

ALS, also known as Charcot’s disease, is a lethal and uncommon neurodegenerative disorder of the central nervous system (143). Early-stage symptoms are often misdiagnosed due to their similarity to other conditions, resulting in delayed diagnosis (143). ALS manifests through muscle atrophy, spasticity, dysphagia, respiratory insufficiency, and cognitive symptoms including irritability, compulsive behaviors, and depression (143). Despite extensive research, the exact pathophysiological mechanisms underlying ALS remain incompletely understood.

Over thirty genes and loci have been implicated in ALS, including C9orf72, TARDBP, FUS, VCP, PFN1, TBK1 (144). These genetic mutations contribute to the multifaceted nature of ALS, causing disruptions in protein homeostasis, RNA metabolism, DNA repair, excitotoxicity, endosomal/vesicular transport, and neuroinflammation (144). Extensive evidence has demonstrated abnormal DNA methylation patterns in ALS patients, irrespective of age at onset (144).

DNA methylation patterns in ALS patients reveal significant deviations involving multiple genes and biological pathways (145, 146). Early studies identified notable hypermethylation in genes related to calcium dynamics, oxidative stress, and synaptic function, predominantly in non-promoter regions such as introns and cryptic areas. Subsequent research highlighted that DNA repair genes, such as Ogg1, Apex1, Pnkp, and Aptx, exhibit reduced methylation in sporadic ALS, suggesting that alterations in DNA damage accumulation and repair mechanisms are linked to disease progression (147). Whole-genome DNA methylation analyses of monozygotic and triplet twins have identified significant differential methylation in genes like RAD9B and C8orf46, with RAD9B’s altered methylation confirmed in ALS (148). Hypermethylation of C9orf72 is linked to transcriptional repression in ALS/frontotemporal dementia (FTD) patients, potentially serving as a neuroprotective mechanism that mitigates molecular distortions related to hexanucleotide repeat expansions (149). In postmortem central nervous system tissues from ALS patients, particularly in lower motor neurons, reduced levels of 5mC and 5hmC have been observed, correlating with the presence of TDP-43 proteinopathies (150). Compared to controls, ALS patient lower motor neurons exhibit significantly lower levels of these epigenetic markers, suggesting an association between TDP-43 and DNA methylation, while changes in glial cell methylation are minimal and primarily affect lower motor neurons (151).

Recent meta-analyses have identified 45 differential methylation positions (DMPs) across large sample sets, involving 42 genes enriched in pathways related to metabolism, cholesterol biosynthesis, and immunity (152). Additionally, whole-blood methylation studies have uncovered 34 significant DMPs involving 13 genes, including 5 hypermethylated and 29 hypomethylated sites, and 12 differential methylation regions (DMRs) linked to 12 genes (153). Notably, the methylation levels of certain genes are significantly correlated with age of onset and disease duration.

Collectively, these studies underscore the aberrant DNA methylation patterns in ALS patients, encompassing diverse biological functions and disease mechanisms, including DNA repair, immune response, and cholesterol biosynthesis. These findings underscore the pivotal role of DNA methylation in ALS pathogenesis and may offer novel diagnostic and therapeutic avenues.

### 4.5 Alzheimer’s disease

AD is a progressive neurodegenerative condition that severely impairs cognitive and memory functions (154). This decline is due to neuronal loss, formation of neurofibrillary tangles (NFTs) and amyloid plaques, ultimately resulting in neuronal death and tissue atrophy (155). In AD, DNA hypermethylation has been reported in the promoters of genes involved in synaptic plasticity, memory formation, and neuroinflammation, potentially contributing to the cognitive decline and neurodegeneration (156, 157).

DNA methylation significantly influences the formation of neurofibrillary tangles (NFTs) and amyloid plaques in AD (158). Specifically, abnormal DNA methylation patterns contribute to amyloid-beta (Aβ) and phosphorylated tau protein aggregation, thereby fostering plaques and tangles formation (159). Methylation alterations in genes such as sirtuin1 (SIRT1), Ankyrin 1 (ANK1), Ribosomal protein L13 (RPL13), Rhomboids family member 2 gene (RHBDF2), and Cadherin 23 (CDH23) have also been implicated in AD pathogenesis, influencing Aβ and tau pathways (**Table 2**). Aberrant DNA methylation patterns may contribute to the onset and progression of AD (160). Furthermore, research has demonstrated that reduced levels of 5mC and its oxidized form 5hmC, are inversely correlated with the accumulation of amyloid-beta (Aβ) and phosphorylated tau proteins in AD (161). The amyloid precursor protein (APP) gene encodes the precursor protein that is cleaved to produce Aβ peptides, which accumulate in the brains of AD patients (162). Hypomethylation of the APP gene may lead to increased expression of APP and enhanced production of Aβ peptides, contributing to the development of AD pathology (163). Age-related memory loss is tied to changes in DNA methylation patterns, which are modulated post-learning (164, 165). Modulating Dnmt3a or Tet2 expression has shown potential in reversing age-related memory impairments (166).Tau proteins, essential in AD DNA methylation, are instrumental in regulating genes linked to Aβ and tau pathways (167). Target genes involved in AD includes ATP6V1G2 and VCP, hinting at their role in disease progression (168). Moreover, emerging studies highlight sex-specific epigenetic mechanisms in AD, revealing gender-specific correlations with the disease for CpG sites in genes like TMEM39A and TNXB (169).

**Table 2:** DNA methylation and Alzheimer’s disease.

Gene/ protein	Function	Mechanism	Reference
Tau	Protecting from AD	Regulating microtubules and neuronal structure, promoting NFTs formation.	(159)
VCP	Impacting AD progression	Methylation at specific CpG sites, impacting cellular maintenance and functions, including autophagy, chromatin remodeling and DNA repair	(168)
LST1	Enhancing inflammation in AD	Negative regulatory function in leukocyte signaling	(168)
APOE*ϵ4	Genetic risk factor related to AD risk	Promoting Aβ accumulation	(238)
TMEM39A	Genetic markers related to AD risk	Affecting sex-specific susceptibility	(169)
TNXB	Genetic markers related to AD risk	Affecting sex-specific susceptibility	(169)

Reactive oxygen species (ROS) and DNA methylation are crucial in AD pathogenesis. ROS, produced during normal metabolism, increase under oxidative stress, causing damage to lipids, proteins, and DNA (170). This damage accelerates the accumulation of amyloid beta (Aβ) plaques and NFTs, advancing neuronal damage and disease progression. Hydroxyl radicals (•OH), a highly reactive form of ROS, are especially damaging to neuronal DNA, contributing to neurodegeneration in AD (171). Under oxidative stress, DNA methyltransferases DNMT1 and DNMT3B, along with sirtuin SIRT1, relocate to CpG islands (CGIs) and form complexes with PRC2 subunits like EZH2 and EED, leading to localized DNA methylation (172). This primarily affects low-expression regions with high CpG density (HCP), while high-expression CGI promoters resist methylation, though the reasons for this are unclear (172). Additionally, pro-inflammatory cytokines like IL-1β, released by activated microglia, promote Aβ production (173). Nicolia et al. found that IL-1β gene promoter hypomethylation occurs early in AD, with no significant change in late-stage AD compared to controls (173). However, IL6 methylation decreases in the frontal cortex as AD progresses, explaining the observed changes in IL-1β and IL6 protein levels (174).

The finding from the comprehensive AD epigenome-wide association study (EWAS) described 5246 CpG sites and 832 differentially methylated regions in 296 brain samples highlights the widespread changes in DNA methylation associated with AD (175). The study identified significant associations between the CpG site cg08806558 at the IGF1 promoter and AD features, such as Braak stages and amyloid plaques, suggesting IGF-1’s role in β-amyloid clearance (175). It confirmed known AD-related CpG sites and discovered new associations, including GPR56 (175). The findings highlight a subnetwork involved in insulin-like growth factor transport regulation, revealing its potential role in AD (175). Furthermore, the effective discrimination of AD and non-demented controls based on multiple CpG sites and RNA expression underscores the potential of DNA methylation patterns as biomarkers for AD diagnosis and prognosis (175). DNA methylation in blood and brain samples is emerging as a vital biomarker for AD. The hypermethylated APP gene, in particular, stands out for AD prognosis (53). Pioneering studies indicate that targeting DNMTs could mitigate amyloid pathology (176). Compounds like epigallocatechin gallate and etanercept show potential in AD treatment (176). Essential methylation compounds, including vitamin B, folic acid, and SAMe, are currently under clinical scrutiny for their therapeutic potential in neurodegenerative diseases (177–179).

These findings underscore the critical role of DNA methylation and hydroxymethylation in regulating the expression of genes involved in AD pathogenesis. Nonetheless, targeted DNA methylation holds potential as a therapeutic approach for reducing Aβ levels in AD, which may have implications for the potential therapeutic targets to mitigate AD pathology.

### 4.6 Parkinson’s disease

PD manifests as a constellation of symptoms, encompassing motor retardation, postural instability, tremor, and cognitive impairment, predominantly affecting the aged (180–182). High levels of dopamine metabolism, iron, and calcium, coupled with mitochondrial dysfunction and neuroinflammation, contribute to the excessive production of ROS in the brains of PD patients (183). This increased oxidative stress damages dopaminergic neurons and exacerbates neurodegeneration. Reports indicate that DNA methylation changes in genes related to dopaminergic signaling, mitochondrial function, and neuroinflammation are present in PD patients (183). For example, cytoplasmic α-synuclein (SNCA) methylation leads to α-synuclein accumulation and ROS production, while PARK7 (DJ-1) hypomethylation impairs antioxidant function (184). These alterations may contribute to the loss of dopaminergic neurons and motor symptoms characteristic of PD (185–187).

Recent epigenomic assessments have unearthed specific DNA methylation aberrations in genes pivotal to PD, such as SNCA (188), Transmembrane and coiled-coil domain family 2 (TMCC2) (189), Solve Carrier Family 17 Member 11 (SLC7A11) (190), HOX transcript antisense RNA (HOTAIR) (191), SLC17A6 (VGLUT2), PARK7 (DJ-1), PTPRN2 (IA-2β), and NR4A2 (NURR1) (192) (**Table 3**). For instance, SNCA intron 1 methylation modulates its transcription and has been directly implicated in PD pathology (188, 193). The methylome status of TMCC2 has been linked with Braak Lewy body staging in dementia and PD (189). Emerging evidence also implicates elevated SLC7A11 methylation, which perturbs glutathione levels, in PD pathogenesis (194). Epigenetic dysregulation in PD is further illustrated by the hypermethylation of the PGC1-α promoter, a master regulator of mitochondrial biogenesis (27, 195, 196). Additional studies indicate that HOTAIR overexpression in PD facilitates SSTR1 gene methylation, worsening motor function and dopaminergic neuron survival (191). Gene-specific hypomethylation in PARK7 (DJ-1) and NR4A2 (NURR1) suggests their involvement in both idiopathic and familial forms of PD (197–200).

**Table 3:** DNA methylation and Parkinson’s disease (PD).

Gene/ protein	Function	Mechanism	Reference
SNCA	Contributing to PD pathology	Increasing accumulation of α-synuclein and ROS production, affecting dopaminergic neuron function	(193)
TMCC2	Contributing to PD pathology	Forming complexes with APOE and APP, regulating to Braak Lewy body staging	(189)
SLC7A11	A biological target in PD	Transporting cystine and glutamate, disrupting glutathione levels and increasing oxidative stress	(190)
HOTAIR	Promoting neurodegeneration in PD	Influencing ERK1/2 signaling	(191)
PARK7	Protecting cells from oxidative stress in PD	Impairing antioxidant function and resulting in dopaminergic neuron loss	(192)
SLC17A6	Transporting glutamate in PD	Altering neurotransmitter release and vesicular transport	(192)
PTPRN2	Protecting from PD	Impacting the function of insulin signaling-related proteins	(192)
NR4A2	Protecting from PD	Affecting the development and function of dopaminergic neurons	(192)

Indeed, compounds such as 5-aza-cytidine and RG108, which are designed to target DNA methylation, are currently being investigated for their potential efficacy in neurodegenerative diseases, such as PD (201). These compounds work by inhibiting DNA methyltransferases and promoting DNA demethylation, potentially reversing abnormal DNA methylation patterns associated with PD (202–204). However, these compounds also have limitations. 5-aza-cytidine can cause side effects such as nausea, vomiting, appetite loss, and bone marrow suppression, and its complex metabolic process may affect its efficacy (205). RG108’s effectiveness is still under investigation, with issues related to its stability and bioavailability potentially impacting its effectiveness (205). A comprehensive understanding of the epigenetic foundations of PD could enable clinicians to tailor treatment regimens to individual methylation profiles, advancing the field of precision medicine (205). By targeting specific DNA methylation changes, personalized therapeutic approaches for PD may become a reality (206).

### 4.7 Huntington’s disease

HD, an autosomal dominant neurodegenerative disorder, is characterized by choreiform movements, mental health disturbances, and cognitive decline (207). The pathology predominantly manifests in the striatal region, affecting various brain areas and causing multifaceted neural impairments in motor control, cognitive abilities, and emotional balance (208). Oxidative stress and mitochondrial dysfunction are intricately linked and work in tandem within the pathology of HD, playing crucial roles in the disease’s mechanisms and progression (209).

The etiology of HD is primarily attributed to the huntingtin protein (HTT) gene contains an abnormal number of CAG repeats, leading to the amplification of trinucleotide repeat sequences (TNR), specifically the CAG codon, resulting in the synthesis of an abnormal protein with an expanded polyglutamine (polyQ) tract (210, 211). In investigating DNA methylation changes in cells expressing mutated HTT, the researchers utilized a technique called Reduced Representation Bisulfite Sequencing (RRBS) to map DNA methylation sites in cells with either wild-type or mutant HTT (212). RRBS, which enriches CpG-rich regions of the genome, provides high-resolution methylation data for these areas. The method involves digesting genomic DNA with a restriction enzyme targeting CpG sites, followed by bisulfite treatment to convert unmethylated cytosines to uracils, and subsequent sequencing of the treated DNA (212). The study revealed that, in the presence of mutant HTT, a significant portion of genes with altered expression exhibited pronounced changes in DNA methylation, particularly in regions with low CpG content known to undergo methylation changes due to neuronal activity. Further analysis identified AP-1 and SOX2 as key transcriptional regulators associated with these DNA methylation changes, a finding validated through genome-wide chromatin immunoprecipitation sequencing (ChIP-seq) (212). Notably, alterations in the binding of AP-1 family members, such as FRA-2 and JUND, were closely linked to increased DNA methylation, suggesting that mutant HTT may disrupt normal transcriptional regulation (212). These insights elucidate how mutant HTT affects neurogenesis by altering DNA methylation patterns and highlight new research avenues and therapeutic strategies for early intervention in HD.

Additionally, an investigation observed a significant reduction in 5-hydroxymethylcytosine (5-hmC) signals in the brain tissue of transgenic mice carrying an artificial chromosome with 128 CAG repeat sequences (YAC128 mice), indicating impaired 5-hmC reconstruction in the brains of HD mice after birth (213). The study also identified disease-specific hydroxymethylation regions (DhMRs) associated with positive epigenetic regulatory factors influencing gene expression, such as axonal guidance signaling, GABA receptor signaling, and Wnt/β-catenin/Sox, affecting neuronal development and function in HD pathogenesis (213). A large-scale DNA methylation study further confirmed the link between HD and an increase in epigenetic age in human blood DNA, with the accelerated epigenetic aging positively associated with the progression of motor symptoms (214).

Advances in technologies and research approaches, such as high-throughput sequencing, single-cell analysis, and genome editing, are providing new opportunities to study DNA methylation and its role in HD more comprehensively. This deeper understanding could pave the way for novel therapeutic strategies aimed at modulating DNA methylation patterns to mitigate HD pathogenesis.

### 4.8 Fragile X syndrome

FXS is the most prevalent inherited intellectual disability, emerging early in life with a range of impairments including deficits in communication skills, cognitive abilities, physical features, as well as epilepsy, anxiety, and heightened sensitivity to stimuli (215). Associated with the Fragile X Mental Retardation 1 (FMR1) gene, FXS is the second most prevalent cause of comorbid autism spectrum disorders (216). The primary etiology of FXS involves the expansion of CGG trinucleotide repeats in the 5′ untranslated region of the FMR1 gene, typically ranging from 55 to 200 repeats in premutation carriers and exceeding 200 repeats in full mutation alleles (216). This expansion leads to FMR1 promoter silencing via an epigenetic mechanism involving DNA methylation of the CGG repeat region and adjacent regulatory areas (216).

The core pathology of FXS revolves around the expansion of CGG repeats within the FMR1 gene, which triggers DNA methylation and subsequent gene silencing (216). Specifically, when the CGG repeat count exceeds 200, DNA methylation at the FMR1 promoter increases. This methylation inhibits transcription factor binding and converts chromatin into a more condensed, transcriptionally inactive state, leading to FMRP deficiency and the manifestation of FXS symptoms (216). Further research indicates that with more than 400 CGG repeats, DNA methylation and gene silencing become significantly more stable, demonstrating a more persistent methylation state (216).

The expanded CGG repeats can form stable secondary structures, such as hairpins, which disrupt normal transcriptional processes (217). These structures, coupled with DNA methylation and histone modifications, make reactivation of the FMR1 gene challenging (217).

In response to these epigenetic changes, several therapeutic strategies have been explored. 5-azacytidine and 5-azadeoxycytidine have been used to remove DNA methylation and restore FMR1 gene transcriptional activity (218). These drugs have shown partial recovery of FMRP expression in FXS cell lines, but their effects are transient, and issues such as cytotoxicity and lack of cell specificity persist (218). Histone deacetylase inhibitors, like 4-phenylbutyrate and sodium butyrate, can induce histone acetylation but show limited effects when used alone (218). They exhibit synergistic effects when combined with demethylating agents, offering more effective restoration of FMR1 gene activity (218).

CRISPR/Cas9 gene-editing technology offers a promising approach for FXS treatment by excising CGG repeat expansions or specifically editing DNA methylation. This technology has demonstrated the potential to restore FMR1 gene function and improve electrophysiological abnormalities in neurons (219, 220). These findings suggest that targeting DNA methylation and histone modifications could partially restore FMR1 gene function and provide potential therapeutic pathways for FXS (219, 220). However, further research is required to address the limitations of current therapies and develop more effective, specific treatments.

Additionally, research into pharmacological interventions has aimed at correcting synaptic defects and restoring FMR1 gene activity. Investigations into epigenetic interventions, particularly DNA demethylation, have included the use of 5-azacytidine and 5-azadeoxycytidine (221). These treatments have achieved partial restoration of FMR1 activity *in vitro*, though the effects are temporary and constrained to the FMR1 promoter region (222). The toxicity of 5-azadC limits its clinical application, but combining it with histone deacetylase inhibitors like sodium butyrate has shown synergistic effects in FXS lymphoblastoid cell lines, leading to more effective restoration of FMR1 gene activity. These studies highlight the potential of targeting DNA methylation and histone modifications to restore FMR1 function, though further research is necessary to develop more effective and less toxic treatment strategies.

### 4.9 Epilepsy

Epilepsy is a complex neurological disorder, primarily classified into focal epilepsies (such as mesial temporal lobe epilepsy, MTLE), generalized epilepsies, and secondary epilepsies (223). Characterized by recurrent seizures, epilepsy significantly impacts cognitive, psychological, and social functioning (223). The pathophysiological mechanisms of epilepsy vary across different types, but epigenetic studies have highlighted the pivotal role of DNA methylation in multiple forms of the disorder.

In temporal lobe epilepsy (TLE), reelin gene hypermethylation disrupts the dispersion of granule cells and the expression of reelin (224, 225). Key epigenetic factors such as NEUROD2 and MECP2 play significant roles in epilepsy; the former is involved in DNA demethylation, while the latter regulates neurodevelopment and synaptic function (226). Methylation changes in the BDNF gene promoter are associated with drug-resistant epilepsy, whereas DNA methylation alterations in the SCN1A and SCN2A promoters are linked to Dravet syndrome (227, 228).

Mesial temporal lobe epilepsy (MTLE), often accompanied by hippocampal sclerosis (HS), is characterized by severe neuronal loss and glial proliferation in the hippocampus (229, 230). In MTLE, DNA methylation levels exhibit a significant negative correlation with gene expression. Notably, the variability of DNA methylation in the hippocampus is higher than in the cortical regions, which may be closely related to the severe neuronal damage in the hippocampus (229, 230). The changes in DNA methylation primarily involve pro-inflammatory mechanisms, such as MHC class II antigen presentation, suggesting a pivotal role of neuroinflammation in the progression of MTLE (231). Alterations in DNA methylation of microglia may affect their inflammatory responses, thereby influencing the course of epilepsy.

In the study of drug-resistant temporal lobe epilepsy (DR-TLE), DNA methylation patterns exhibit significant changes across different brain regions and peripheral blood. Analysis using the Illumina Infinium Methylation EPIC BeadChip array on 19 DR-TLE patients and 10 non-epileptic controls revealed 32, 59, and 3,210 differentially methylated probes (DMPs) in the hippocampus, amygdala, and epileptogenic zone surrounding cortex (SCEZ), respectively (232). These DMPs are associated with genes involved in neurotrophic factors, calcium signaling, voltage-gated channels, and inflammatory processes.

Particularly noteworthy is the analysis of cell-free DNA (cfDNA) methylation, which provides a non-invasive means to reflect disease mechanisms. In DR-TLE, cfDNA methylation patterns show significant overlap with those observed in the hippocampus, suggesting that cfDNA could serve as a potential peripheral biomarker for the disease (233). The Illumina Infinium Methylation EPIC BeadChip analysis revealed substantial differences in genome-wide DNA methylation patterns between MTLE patients and healthy controls, including genes involved in anion binding, redox activity, and cell growth regulation, such as SLC34A2, CLCN6, and CYP3A4 (234).

Notably, the DMP (cg26834418, CHORDC1) in the hippocampus demonstrates a strong blood-brain correlation, indicating its potential as a peripheral biomarker for DR-TLE (232). Additionally, the methylation status of several DMPs in the SCEZ (such as SHANK3, SBF1, and MCF2L) was validated through methylation-specific qPCR (232). The differentially methylated CpGs were categorized into differentially methylated regions (DMRs): 2 in the hippocampus, 12 in the amygdala, and 531 in the SCEZ. Furthermore, new genes previously unassociated with DR-TLE, such as TBX5, EXOC7, and WRHN, were identified (232). These findings offer novel insights into the epigenetic modifications associated with DR-TLE and suggest that DMPs in the SCEZ may be related to voltage-gated channel in the amygdala.

In summary, these studies reveal the crucial role of DNA methylation in the onset and progression of epilepsy, particularly through the modulation of inflammatory responses. The integration of cfDNA methylation analysis adds a non-invasive dimension to understanding and monitoring the disease. These findings offer new perspectives on the pathological mechanisms of epilepsy and lay a foundation for the development of personalized treatment strategies. Future research should focus on further elucidating the mechanisms of DNA methylation, developing novel diagnostic tools, and advancing personalized therapeutic approaches.

## 5 Conclusion

Significant studies have been made in understanding how DNA methylation modulates gene expression in neurological disorders, yet translating these insights into clinical therapies remains a significant challenge. A challenge exists in the analysis of DNA methylation and its application in disease diagnosis. DNA methylation patterns can vary across different tissues and cell types, making it necessary to obtain samples from the disease-affected tissue for accurate analysis. To overcome these limitations, researchers are exploring alternative approaches, such as analyzing DNA methylation patterns in specific cell types or using tissue-specific markers to enrich for disease-affected cells. Additionally, advancements in technologies, such as single-cell DNA methylation sequencing, may provide more precise and informative insights into tissue-specific DNA methylation patterns. Studies have highlighted notable commonalities in DNA methylation abnormalities across various neurological disorders, underscoring the pivotal role of DNA methylation in disease mechanisms by modulating gene expression and participating in disease mechanisms. Common mechanisms, such as disrupted gene regulation and abnormal inflammatory responses, provide critical insights into disease pathogenesis and may aid in developing a mechanistic model based on DNA methylation. Targeting DNA methylation holds promise for therapeutic interventions. DNA methylation abnormalities are increasingly recognized as diagnostic and prognostic markers, particularly in pathways related to oxidative stress and neuroinflammation. Furthermore, advancements in technologies such as WGBS, RRBS, MSP, methylation arrays, MeDIP-seq, and pyrosequencing have deepened our understanding of the complex DNA methylation landscapes associated with these disorders. However, translating these insights into clinical practice remains challenging due to the complexity of methylation patterns and the need for disease-specific sample analysis.

Despite advances, significant knowledge gaps and clinical challenges remain. Future research should focus on elucidating DNA methylation mechanisms across different diseases, developing reliable diagnostic tools, and formulating effective therapeutic strategies. Specifically, targeting DNA methylation in neurodegenerative contexts requires careful consideration of potential risks, such as side effects and long-term safety concerns. Continued exploration of DNA methylation’s role in neurological disorders could refine diagnostic and prognostic methods and open new avenues for innovative therapeutic interventions.

## Glossary

AD: Alzheimer’s Disease
NFTs: Neurofibrillary Tangles
Ab: Amyloid-beta
SIRT1: Sirtuin 1
ANK1: Ankyrin 1
RPL13: Ribosomal Protein L13
RHBDF2: Rhomboids Family Member 2
CDH23: Cadherin 23
5mC: 5-Methylcytosine
5hmC: 5-Hydroxymethylcytosine
APP: Amyloid Precursor Protein
Dnmt3a: DNA Methyltransferase 3A
Tet2: Ten-Eleven Translocation2
ATP6V1G2: ATPase H+ Transporting V1 Subunit G2
VCP: ValosinContaining Protein
TMEM39A: Transmembrane Protein 39A
TNXB: Tenascin XB
ROS: Reactive Oxygen Species
OH: Hydroxyl Radicals;
DNMT1: DNA Methyltransferase 1
DNMT3B: DNA Methyltransferase 3B
PRC2: Polycomb Repressive Complex 2
EZH2: Enhancer of Zeste 2
EED: Embryonic Ectoderm Development
IL-1b: Interleukin-1 Beta
IL6: Interleukin 6
EWAS: Epigenome-Wide Association Study
GPR56: G Protein-Coupled Receptor 56
5-aza-cytidine: 5-Azacytidine
RG108: A DNA Methyltransferase Inhibitor
PD: Parkinson’s Disease
SNCA: Synuclein Alpha
PARK7: Parkin 7
DJ-1: Parkinsonism Associated Deglycase
SLC7A11: Solute Carrier Family 7 Member 11
HOTAIR: HOX Transcript Antisense RNA
SLC17A6: Solute Carrier Family 17 Member 6
PTPRN2: Protein Tyrosine Phosphatase Receptor Type N2
NR4A2: Nuclear Receptor Subfamily 4 Group A Member 2
PGC1-a: Peroxisome Proliferator-Activated Receptor Gamma Coactivator 1-alpha
FXS: Fragile X Syndrome
FMR1: Fragile X Mental Retardation 1
CRISPR/Cas9: Clustered Regularly Interspaced Short Palindromic Repeats/Cas9
RRBS: Reduced Representation Bisulfite Sequencing
AP-1: Activator Protein 1
SOX2: SRY-Box Transcription Factor 2
5-hmC: 5-Hydroxymethylcytosine
YAC128: Yeast Artificial Chromosome 128
DhMRs: Disease-Specific Hydroxymethylation Regions
cfDNA: Cell-Free DNA
DMP: Differentially Methylated Probes
DMR: Differentially Methylated Regions
qPCR: Quantitative Polymerase Chain Reaction
